# Association between adolescent life events and depressive symptoms: data from the Shanghai area

**DOI:** 10.3389/fpsyt.2025.1651770

**Published:** 2025-08-19

**Authors:** Shuzhi Peng, Yan Wang, Xin Huang, Xin Zhang, Juan Ge

**Affiliations:** College of Health Management, Shanghai Jian Qiao University, Shanghai, China

**Keywords:** adolescents, depressive symptoms, life events, family factors, nonlinear relationship

## Abstract

**Objective:**

To explore the relationship between adolescent life events and depressive symptoms and the influence of family factors.

**Methods:**

Based on the data of 766 adolescents in Shanghai, this study collected information by electronic questionnaire, and evaluated it by using the Adolescent Life Event Scale (ASLEC) and the Center for Epidemiologic Studies Depression Scale (CES-D).

**Results:**

The study found that there was a significant nonlinear relationship between life event scores and depressive symptoms, and two key inflection points were identified, 13 and 45.5. Teenagers with depressive symptoms are more likely to report poor family economic status and low education level of their mothers.

**Conclusion:**

Family factors play an important role in adolescent depression symptoms, and the research results provide a basis for preventing adolescent depression. In the future, it is necessary to expand samples and longitudinal studies to further verify the conclusions.

## 1 Introduction

Adolescent depressive disorder has evolved into a global public health crisis. Some studies show that the incidence of the disease among people aged 12-18 shows a significant upward trend (1). As a highly disabling psychological disorder, depression not only causes serious damage to individual social function, but also causes heavy social and economic burden (2). Evidence-based medical research has confirmed that the pathogenesis of depression involves multiple interactions between genetic susceptibility, psychological stress, environmental exposure and biomarkers (3–5). It is worth noting that life events, as typical environmental stressors, have a significant impact on young people in the critical period of psychological development. Empirical research shows that negative stressors (such as academic stressors, family system conflicts, etc.) constitute a significant incentive for depressive symptom groups, which enhance individual psychological vulnerability through neuroendocrine mechanisms (6–8). Interdisciplinary research has confirmed that the exposure of life events is positively correlated with the score of depressive symptoms, and the intensity of this correlation has the adjustment effect of individual heterogeneity (9).

The concept of Life Events comes from the stress theory system. Stress refers to an individual’s non-specific physiological and psychological response when faced with internal and external environmental stimuli, which is typically manifested in the activation of the autonomic nervous system, the imbalance of cognitive evaluation and the change of adaptive behavior (10). As a typical psychosocial stressor, life events refer to those remarkable situations that require individuals to mobilize psychological resources for adaptive adjustment and may lead to the reconstruction of social functions, such as academic difficulties, interpersonal conflicts or changes in family structure (11). Empirical research shows that life events are not only the key variables to induce negative emotions, but also have significant correlation with the increase of problem behavior and the occurrence of depression (12, 13). At present, life events, as a stressor with extensive social influence, and their dose-response relationship with depressive symptoms and mediation mechanism have become an important direction of clinical psychology research (14). Teenagers are at the critical stage of their physical and mental development, and their psychological sensitivity to exogenous stress is significantly enhanced. The negative psychological impact caused by life events is easy to produce amplification effect, and then the core symptoms of depression such as depression and lack of interest are induced. The empirical study shows that the risk coefficient of depression in adolescents exposed to life events at high frequency is significantly increased, and the effect has a dose-response relationship. It is worth noting that the establishment of family support system and individual adaptive coping strategies can effectively enhance the psychological resilience of adolescents by enhancing the regulatory function of prefrontal cortex on limbic system, thus achieving a buffering effect on depression risk factors (15).

Studies have proved that life events are positively related to depression, and can predict future depressive symptoms to some extent (16). In adolescence, teenagers face more life events, such as academic pressure, contradictions and conflicts with parents, teachers and classmates, and health problems. Every teenager will experience similar life events, but not every teenager has depression. Previous studies have found that individual qualities such as self-esteem, self-efficacy, negative automatic thinking, coping style and cognitive emotional adjustment can all have an impact on depression (17–19). Based on the data of adolescents in Shanghai, this study deeply analyzes the specific influence of life events on depression symptoms, aiming at providing empirical support for preventing and intervening adolescent depression.

## 2 Materials and methods

### 2.1 Data entry and study population

**Figure 1:**
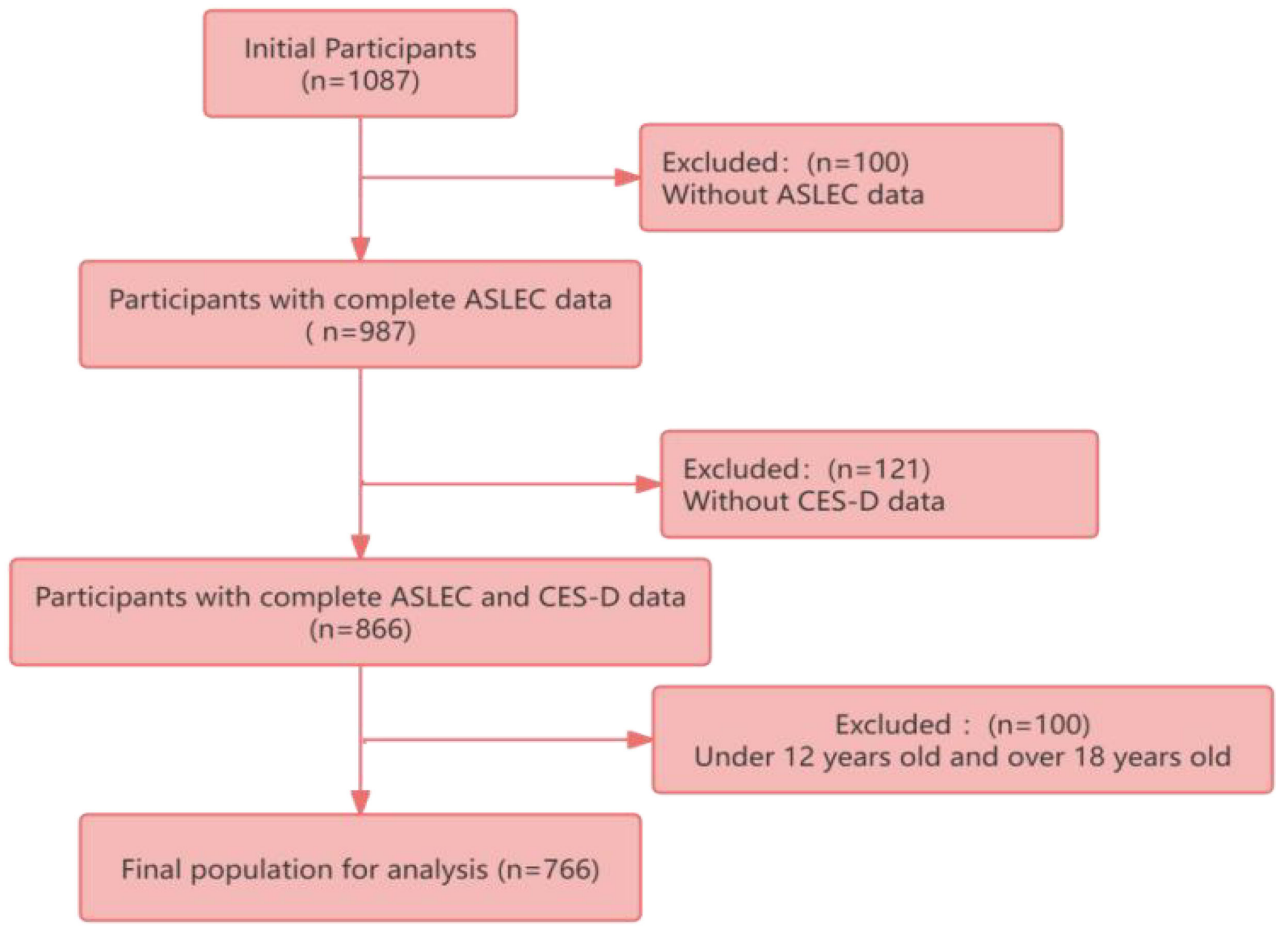
Flow chart of participant selection.

We set up an electronic questionnaire on the questionnaire website. The questionnaire was distributed in the form of WeChat link. Questionnaires were distributed by uniformly trained investigators. A total of 1,100 questionnaires were distributed and 1,087 questionnaires were collected. The quality controller checked the recovered questionnaire in time, and reported the problems to the investigator for verification and correction. Among them, 100 people without ASLEC data and 121 people without CES-D data were under 12 years old and over 18 years old, and the total number of people included in the analysis was 766. The flow chart of participant screening is shown in **Figure 1**.

### 2.2 Life events, definition of depression

Life events are a common stressor, which occurs in the living environment such as family, study and work, and require individuals to make changes or changes such as academic frustration, interpersonal tension and family changes (20). Life events are an important factor that causes negative emotions, which is not only a reason for the increase of problem behaviors, but also an important inducement for depression. The influence degree can be quantitatively evaluated by the Adolescent Self-Rating Life Events Checklist (ASLEC) (21). The higher the score, the greater the stress of life events experienced by individuals. In the process of growing up, teenagers often lack coping experience when facing the pressure of life events, and are easy to fall into emotional difficulties (22). They need support from family, school and society to strengthen psychological resilience and learn positive coping strategies (23). Early identification and intervention can effectively prevent the deterioration of depressive symptoms and help teenagers to establish a healthy psychological defense mechanism, so as to better adapt to various challenges in life.

Depression is an emotional disorder characterized by extreme depression, lack of vitality and slow speech movements. Depression is different from depression (24). Depression is a kind of negative emotion that individuals show when they face external pressure and can’t cope with it (25). When adolescents are depressed, if effective prevention and intervention measures are not taken, their depressed mood is likely to deteriorate and even develop into depression, which will bring great harm to the physical and mental development of adolescents (26). At present, there are about 95 million people suffering from depression in China, and about 280,000 people commit suicide every year, and about 40% of them die of depression. For teenagers, its harm is mainly manifested in the decline of academic performance, the damage of interpersonal relationships such as family and peers, the development of destructive behavior, the increase of suicidal intention and the possibility of suicidal behavior, and substance abuse (27). More importantly, depression has a high recurrence, which not only affects the physical and mental health of individuals, but also increases the disease burden of families. Depressive symptoms were judged according to the Center for Epidemiology’s Depression Scale (CES-D), and when the score reached or exceeded the critical value, it was considered that there were depressive symptoms (28).

### 2.3 Measuring tools

#### 2.3.1 Adolescent life events measurement

Life event measurement adopts the Adolescent Self-Rating Life Events Scale (29). The self-rating scale includes 27 items, covering life events that may have a negative impact on teenagers’ physiology and psychology, such as “losing face in public” and “fighting with others”. It mainly includes six dimensions, namely, interpersonal relationship, loss, punishment, learning, health and others. Interpersonal relationship includes 1 question, 2 questions, 4 questions, 15 questions and 25 questions, loss includes 12 questions to 14 questions, punishment includes 17 questions to 21 questions, 23 questions and 24 questions, and learning includes 3 questions, 9 questions, 16 questions, 18 questions and 22 questions (30). According to their psychological reaction, the subjects made a five-level evaluation, that is, no influence, mild, moderate, severe and extremely heavy. The observation index was the sum of the total scores of each dimension, and the total score was calculated by adding the total scores of each dimension (31). The higher the score, the greater the influence of life events on individuals. The reliability of the scale is 0.85, the split-half reliability is 0.88, the average correlation coefficient of each event in the retest reliability is 0.5, and the correlation coefficient of the total score is 0.69 (32).

#### 2.3.2 Measurement of depressive symptoms

The depression scale adopts the CES-D compiled by Radloff of the National Institute of Mental Health, which is mainly used to evaluate the depressive symptoms in the current week, focusing on depressed emotions or depressed mood (33). The scale is a 20-item self-rating scale, with a score of 4 (0-3), in which the 4th, 8th, 12th and 16th items are scored in reverse, and the sum of all items is the total score of the scale, in which the total score ≤9 is no depressive symptoms, and > 9 is depressive symptoms, and the higher the score, the higher the degree of depression. CES-D scale has good internal consistency validity and structural validity in different cultural backgrounds (34).

### 2.4 Statistical analysis

Continuous variables are expressed as mean standard deviation, while classified variables are expressed as proportion. T test was used to compare the differences between the two groups for continuous variables. The classified variables were compared by chi-square test. Logistic regression model was used to analyze the correlation between life event score and depression. Subgroup analysis was used to confirm the stability of the association between life event scores and depression in different subgroups. RCS was chosen to capture potential nonlinear thresholds in life event effects, which linear models might miss. Knots were determined via Akaike Information Criterion (AIC) optimization and visual inspection of residual plots. R software (version 4.3.3) was used in all the analyses.

## 3 Results

### 3.1 Baseline characteristics of the study population

Baseline characteristics of the study participants are presented in **Table 1**. The study included 766 participants, of whom 202 (26.4%) reported depression symptoms. Participants with depression were significantly more likely to report poor family financial status (p < 0.01) and had mothers with lower educational attainment (junior high or less; p = 0.028) compared to those without depression. No significant differences were observed between the groups regarding sex, age, ethnicity, domicile (country/city), overall education level, boarding school status, being an only child, living arrangements with parents, or father’s educational level (all p-values > 0.05). The vast majority of participants (95.2%) attended boarding school. Notably, paternal education level showed no significant difference between depressed and nondepressed groups (p = 0.503).

**Table 1:** Baseline characteristics of the study participants.

Characteristics	Overall	Depression		*P*-value
		Without	With	
n	766	564	202	
Sex				0.623
Male	386 (50.4%)	281 (36.7%)	105 (13.7%)	
Female	380 (49.6%)	283 (36.9%)	97 (12.7%)	
Age, years	17.65 ± 0.847	17.62 ± 0.860	17.71 ± 0.810	0.228
Ethnicity				0.611
Han Chinese	479 (62.5%)	356 (46.5%)	123 (16.1%)	
Other ethnicity	287 (37.5%)	208 (27.2%)	79 (10.3%)	
Domicile				0.869
Country	422 (55.1%)	312 (40.7%)	110 (14.4%)	
City	344 (44.9%)	252 (32.9%)	92 (12.0%)	
Education				0.655
Junior high or lower	9 (1.2%)	6 (0.8%)	3 (0.4%)	
High school	179 (23.4%)	13 (17.8%)	43 (5.6%)	
University or above	578 (75.5%)	422 (55.1%)	156 (20.4%)	
Boarding school				0.444
No	37 (4.8%)	25 (3.3%)	12 (1.6%)	
Yes	729 (95.2%)	539 (70.4%)	190 (24.8%)	
Only child				0.776
No	577 (75.3%)	423 (55.2%)	154 (20.1%)	
Yes	189 (24.7%)	141 (18.4%)	48 (6.3%)	
Living with parents				0.471
No	41 (5.4%)	28 (3.7%)	13 (1.7%)	
With father or mother	86 (11.2%)	60 (7.8%)	26 (3.4%)	
With parents	639 (83.4%)	476 (62.1%)	163 (21.3%)	
Family financial				<0.01
Bad	101 (13.2%)	17 (2.2%)	84 (11.0%)	
Average	467 (61.0%)	408 (53.3%)	59 (7.7%)	
Good	198 (25.8%)	139 (18.1%)	59 (7.7%)	
Mother’s education				0.028
Junior high or lower	146 (19.1%)	100 (13.1%)	46 (6.0%)	
Middle school	326 (42.6%)	244 (31.9%)	82 (10.7%)	
High school	134 (17.5%)	110 (14.4%)	24 (3.1%)	
University or above	160 (20.9%)	110 (14.4%)	50 (6.5%)	
Father’s educational				0.503
Junior high or lower	123 (16.1%)	90 (11.7%)	33 (4.3%)	
Middle school	327 (42.7%)	233 (30.4%)	94 (12.3%)	
High school	156 (20.4%)	117 (15.3%)	39 (5.1%)	
University or above	160 (20.9%)	124 (16.2%)	36 (4.7%)	

### 3.2 Analysis of restricted cubic spline regression

**Figure 2:**
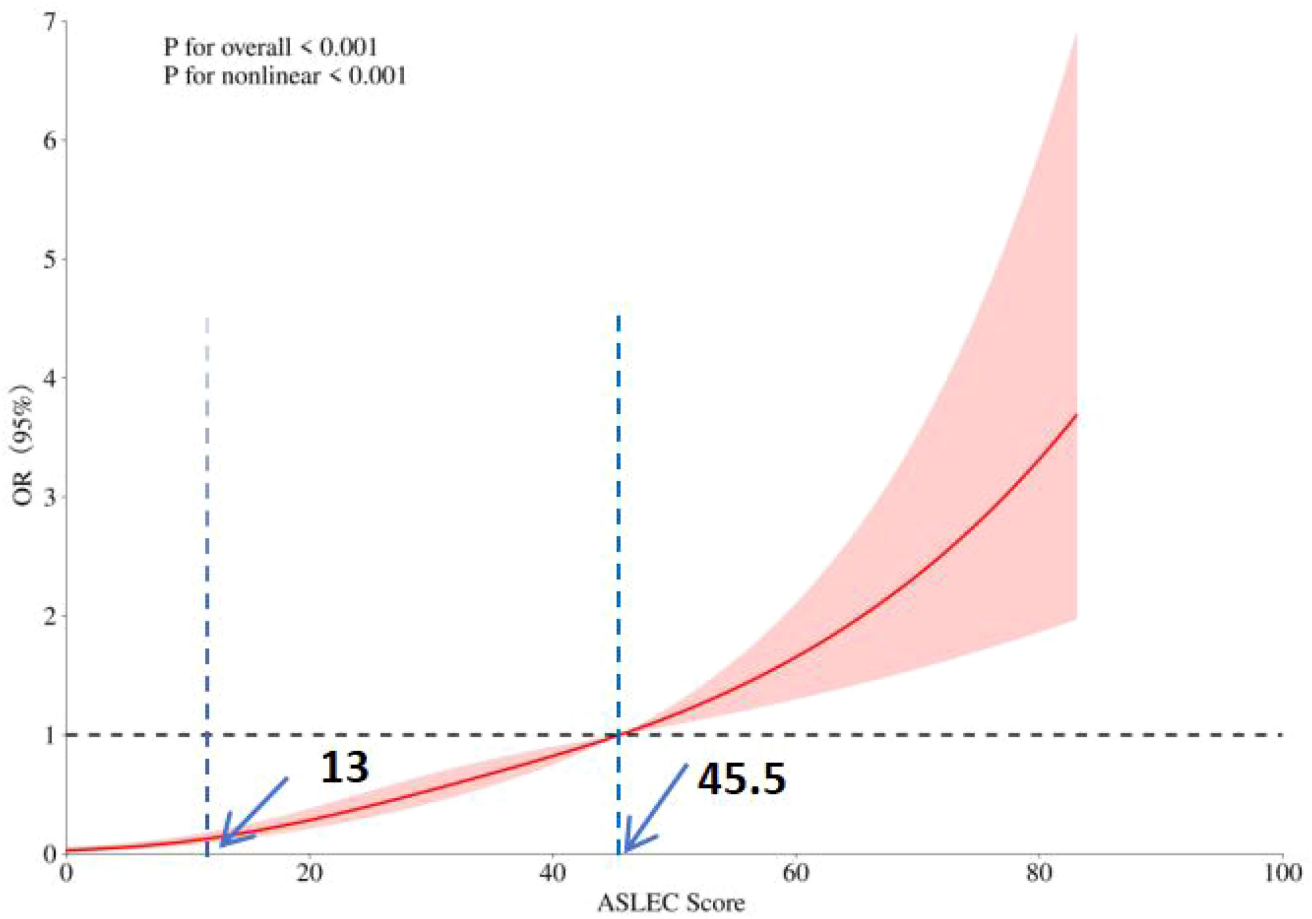
Determination of the association between adolescent life events and depressive symptoms by restricted cubic spline (RCS) regression analysis.

In the restricted cubic spline regression, after adjusting for potential covariates, a significant nonlinear relationship was observed between the ASLEC Score and depressive symptoms (P <0.001) (**Figure 2**). There is a trend of increasing depressive OR with higher ASLEC Scores. The RCS analysis identified two key inflection points: the first at 13 and the second at 45.5. Adolescents with an ASLEC Score below 13 have a very low risk of depressive symptoms. However, adolescents with an ASLEC Score between 13 and 45.5 have a slightly higher risk of depressive symptoms. After the ASLEC Score exceeds 45.5, the risk of depressive symptoms gradually increases. Therefore, it is crucial to keep the ASLEC Score below 45.5 to potentially reduce the risk of depressive symptoms in adolescents.

### 3.3 Association between ASLEC score and depression in different subgroups

In order to determine the stability of the association between ASLEC Score and depressive symptoms, according to the inflection point of ASLEC Score determined by RCS analysis, the whole population was divided into three subgroups, including ≤13, 13–45.5 and ≥45.5. Weighted logistic regression analysis was used to explore the relationship between ASLEC Score (as a classified variable) and the occurrence of depressive symptoms. The results are shown in **Table 2**. Compared with individuals whose depressive symptoms are lower than 13, individuals whose depressive symptoms level are between 13 and 45.5 or whose depressive symptoms level are higher than 45.5 show a significant increase in the risk of depression in both unadjusted and adjusted models (both P values < 0.05 and trends P < 0.05).

**Table 2:** Association between ASLEC Score (as a three categorical variable) and depressive symptoms based on the inflection points identified from RCS analysis.

Variables	Model1		Model2		Model3	
	OR (95%CI)	*P*	OR (95%CI)	*P*	OR (95%CI)	*P*
ASLEC Score						
≤13	1.00 (Reference)		1.00 (Reference)		1.00 (Reference)	
13-45.5	6.79 (4.43 ~ 10.41)	<.001	7.00 (4.55 ~ 10.77)	<.001	7.38 (4.74 ~ 11.50)	<.001
≥45.5	29.17 (15.77 ~ 53.97)	<.001	32.27 (17.08 ~ 60.97)	<.001	33.53 (17.37 ~ 64.76)	<.001

Depressive symptoms were converted from a continuous variable to a three categorical variable based on the inflection points identified from RCS regression analysis. Model 1: no covariates were adjusted; Model 2: adjusted for gender, age, and ethnicity, domicile, education; Model 3: gender, age, and ethnicity, domicile, education, boarding school, only child, living with parents, family financial, mother’s education, father’s educational.

## 4 Discussion

### 4.1 The nonlinear relationship between adolescent life events and depressive symptoms

In this study, through the restricted cubic spline regression analysis, the significant nonlinear relationship between the scores of ASLEC and the depressive symptoms were revealed, and two key inflection points, 13 and 45.5, were identified. This discovery is of great value for understanding the mechanism of adolescent depression symptoms. When the score of ASLEC is lower than 13, the risk of depression symptoms of adolescents is extremely low, which shows that under this life event stress level, the psychological resilience of adolescents is relatively strong, and they can better cope with the stressors in life and maintain psychological stability. However, when the score is between 13 and 45.5, the risk of depressive symptoms increase, indicating that the stress of life events at this stage may begin to have a more obvious negative impact on the mental health of adolescents, and their psychological coping mechanisms may face certain challenges. When the ASLEC score exceeds 45.5, the risk of depressive symptoms gradually increase significantly, which means that life events with too high frequency and intensity are exposed, which is beyond the scope of adolescents’ psychological tolerance and adjustment, thus leading to a significant increase in the risk of depressive symptoms (35). Defining these two inflection points has practical guiding significance for clinical practice and preventive intervention, and can help identify adolescent groups with different risk levels, so as to take corresponding preventive and intervention measures according to different score intervals. Such as strengthening psychological support and coping strategy training for adolescents with scores of 13-45.5. For high-risk adolescents with scores over 45.5, more active psychological intervention and close monitoring are needed to reduce the risk of depressive symptoms, which is also helpful to optimize resource allocation and improve the effectiveness and pertinence of intervention (36).

### 4.2 The role of family factors in adolescent depression symptoms and their interaction with life events

While adolescents exhibiting depressive symptoms demonstrated a higher likelihood of reporting poorer family financial status and lower maternal education levels, paternal education demonstrated no statistically significant association (*p* = 0.503). This finding indicates distinct parental roles within family systems: Maternal education may directly impact emotional support patterns, parenting behaviors, and stress-coping modeling, consistent with attachment theory, which emphasizes the mother-child dyad in emotional regulation development (37). The non-significance of paternal education may be attributable to traditional gender roles within Chinese households, wherein fathers primarily provide financial provision rather than emotional support. Poor family economic status may lead to problems such as lack of living resources, poor living environment, limited educational opportunities, etc. These factors will increase the pressure of life events faced by teenagers, such as academic difficulties and interpersonal tension, and at the same time weaken the psychological support and coping resources provided by families to teenagers, making them more likely to get into trouble when facing life events, and then induce depressive symptoms. However, mothers’ low education level may affect their cognition and coping ability to teenagers’ psychological problems, and they can’t detect the psychological changes of teenagers in time and give effective support and guidance, which further aggravates the negative impact of life events on teenagers’ mental health. This shows that when preventing adolescent depression symptoms, we need to pay attention to the overall situation of the family environment, especially to improve the mother’s education level and family economic support ability. By improving the family environment, we can enhance adolescents’ ability to cope with life events and reduce the risk of depression symptoms, such as carrying out mental health education activities for parents, improving parents’ understanding and coping ability with adolescent psychological problems, and providing corresponding social policy support to improve family economic situation and create a family environment more conducive to the development of mental health for adolescents.

### 4.3 Advantages and limitations

The advantages of this study are mainly reflected in the following aspects: First, the sample size is large, including 766 adolescents, which provides rich data for analyzing the relationship between adolescent life events and depressive symptoms, and helps to improve the stability and representativeness of the results. Secondly, the study adopted standardized measurement tools, including the ASLEC and the CES-D. These tools have good reliability and validity, and can accurately quantify life event stress and depressive symptoms, thus ensuring the reliability of the research results. In addition, a variety of statistical analysis methods, such as restricted cubic spline regression and logistic regression, are used, which not only deeply analyzes the nonlinear relationship between them, but also determines the key inflection point, and verifies the stability of the results through subgroup analysis, making the research conclusion more scientific and rigorous (38). At the same time, based on the data of adolescents in Shanghai, this study provides empirical support and theoretical basis for the prevention and intervention strategies of regional adolescents’ depressive symptoms, which has important practical value. Finally, the study also pays attention to the influence of family factors on adolescent depression symptoms, which enriches the etiological understanding of adolescent mental health. Although this study provides valuable insights into the relationship between adolescent life events and depressive symptoms, there are still some limitations that need to be further improved in future research (39). First of all, the research sample mainly comes from boarding school students in Shanghai, which may limit the universality of the research results. As an economically developed city, Shanghai has certain particularities in the living environment, educational resources and family background of its teenagers, which may be quite different from other regions, especially those with relatively backward development. Therefore, the future research should expand the sample range, covering different areas, different types of schools and different social and economic backgrounds, in order to verify the wide applicability of the results of this study, and more comprehensively understand the characteristics and differences of the relationship between adolescent life events and depressive symptoms in different groups. Secondly, the cross-sectional design is adopted in this study, and it is difficult to determine the causal relationship and timing between life events and depressive symptoms. Although statistical analysis reveals the relationship between them, it is not clear whether life events directly lead to the occurrence of depressive symptoms, and there are other potential confounding factors that may affect the relationship between them.

### 4.4 Future research direction

In the future, a longitudinal study design can be used to follow up adolescents for a long time and collect multiple measurement data, so as to better explore the causal path between life events and depressive symptoms and the changing trend with time, and further clarify the mechanism of life events in the development of adolescent depressive symptoms. In addition, the specific regulatory mechanism of individual psychological quality in the relationship between life events and depressive symptoms was not discussed in depth, only the regulatory role of individual psychological quality was mentioned. Future research can further introduce more psychological variables, such as self-esteem, self-efficacy, coping style, cognitive emotional adjustment strategies, etc., deeply analyze how these psychological factors play a role in the process of life events affecting depression symptoms, and reveal their internal mechanisms by constructing more complex models, so as to provide more sufficient theoretical basis for formulating more targeted psychological intervention measures, such as developing intervention projects aimed at cultivating adolescents’ psychological quality, improving their ability to cope with life events, and preventing and reducing the occurrence of depression symptoms.

## 5 Conclusion

Depressed teenagers are more likely to have poor family economic status and low education level of their mothers. In the future, it is necessary to expand the sample and longitudinal study to verify the conclusion, so as to formulate accurate intervention strategies and alleviate adolescent depression.

## Data Availability

The raw data supporting the conclusions of this article will be made available by the authors, without undue reservation.
